# Map of biomedical research in Cameroon; a documentary review of approved protocols from 1997 to 2012

**DOI:** 10.1186/s12992-017-0312-y

**Published:** 2017-11-21

**Authors:** Ebile Akoh Walter, Ateudjieu Jerome, Djuidje Ngounoue Marceline, Martin Ndinakie Yakum, Watcho Pierre

**Affiliations:** 1 Department of Biomedical Sciences, University of Dschang, Dschang, Cameroon; 2 Division of Health Operations Research, Ministry of Public Health, Yaoundé, Cameroon; 3 M.A. SANTE (Meilleure Access aux soins de Santé), P.O. Box 33490, Yaoundé, Cameroon; 4 Department of Biochemistry, Faculty of Science, University of Yaoundé I, Yaoundé, Cameroon; 5 Research and Health Ethics Committee in Central Africa “Comité d’Ethique de la Recherche et de la Santé en Afrique Centrale (CERSAC), Yaoundé, Cameroon

**Keywords:** Map, Biomedical research, Cameroon, Documentary review, Protocol

## Abstract

**Background:**

Over the last decade, there has been a rapid increase in biomedical research in Cameroon. However, the question of whether these research projects target major health priorities, vulnerable populations and geographic locations at risk remains to be answered. The aim of this paper is to describe the state of biomedical research in Cameroon which is a key determinant that would guide future health care policies and promote equitable access to healthcare.

**Methods:**

A documentary review of all approved protocols (proposals) of biomedical research projects, from 1997 through 2012, at the Cameroon National Ethics Committee. Protocols were reviewed systematically by independent reviewers and data were extracted on a grid. Data were analyzed by calculating proportions at 95% confidence interval, chi-square test (chi2) and *p*-values.

**Results:**

Two thousand one hundred seventy two protocols were reviewed for data extraction. One thousand three hundred ninety-five (64.7%) were student projects, 369 (17.0%) projects had international sponsors, and 1528 (72.4%) were hospital-based studies. The most targeted domain was the fight against diseases 1323 (61.3%); mostly HIV 342 (25.8%) and Malaria 136 (10.3%). Over half of the studies were concentrated in the Centre region 1242 (57.2%), with the least projects conducted in the Northern region 15 (0.7%). There was strong evidence that international and local sponsors would influence the research site (*p*-value = 0.01) and population targets (*p*-value = 0.00).

**Conclusion:**

Although biomedical research targets some important diseases that pose a great burden to Cameroonians, the most vulnerable populations are excluded from research. Biomedical research scarcely addresses other components of the health system and emerging diseases of vital public health importance. We recommend that the government should play a central role, between researchers from academic institutions, sponsors, NGOs and research institutions, to ensure that biomedical research addresses the health priorities of Cameroonians. It should include vulnerable populations, and address other components of the health system for a balance. These recommendations are critical to ensuring that future research informed health policies reflect the health needs of the populations and promote equity in healthcare access.

**Electronic supplementary material:**

The online version of this article (10.1186/s12992-017-0312-y) contains supplementary material, which is available to authorized users.

## Background

Cameroon is working towards being an emergent country by 2035 [1].The health sector is one of the seven key components, that has been identified to benefit from the 2010–2019 implementation phase of the national development policy, and of the sustainable development strategy [2, 3]. With growing urgency, and the necessity for the government and its partners to improve the health and wellbeing of Cameroonians, a vast array of health programs and health interventions have been employed. In support of that, there has been an increasing need for biomedical research to inform the relevant health policies, health services, and health interventions in a contextual and rational manner with regards to the population choice, site of research, the target diseases or the component of the health system. Over the last decade, there is a rapid increase in the rate of biomedical research projects conducted in Cameroon. However, little or no information is available to determine whether these research projects target Cameroonians health priorities. The aim of this paper is to map or describe the state of biomedical research in Cameroon and the influence of sponsors on the implementation of these research projects. Findings from this study will aid the government, and stakeholders to develop future health policies that will guide biomedical research to reflect the health needs of Cameroonians, promote equity in healthcare access, and strengthen the Cameroon health system as a whole.

This study was conducted at the Cameroon National Ethics Committee (CNEC). Created in 1987 [4], CNEC is the oldest and the only officially recognized competent ethics review committee in Cameroon and abroad. The CNEC is free from all political, institutional, professional, and economic influence. It is one of the major actors/key player/stakeholder involved in health research in Cameroon. It plays a basic role to safeguard the dignity, rights, safety, physical integrity and the well-being of potential participants in biomedical research conducted in Cameroon. It is also a Center of focus to ensure collaboration and networking with other ethics review boards at national and international levels. In effect, It receives protocols from applicants locally and abroad continuously throughout the year for ethical evaluation. The committee meets every two months to evaluate protocols submitted for ethical review by local and international researchers. Once a decision is made, only approved protocols are allowed to conduct research on the Cameroon national territory.

## Methods

### Study design

Approved biomedical research protocols (proposals) were reviewed for data extraction. The Cameroon National Ethics Committee (CNEC) was the study site. Protocols were reviewed systematically by independent reviewers and data from 1997 through 2012 were extracted and analyzed in EPIINFO and Microsoft Excel 2010.

### Selection criteria and data collection procedure

#### Data source

Hard copies of biomedical research protocols were the primary source of data. All biomedical research protocols that were submitted for ethical review from January 1997 through December 2012 were included. Non-biomedical research protocols were excluded.

#### Data extraction process

Data were extracted and recorded on a grid that was conceived and tested prior to data extraction. The review process targeted the titles, abstracts, objectives, and methodology of each proposal. Data extraction was systematic, involving four independent reviewers who simultaneously reviewed separate copies of identical protocol, for data extraction on identical separate datasheets(grids). At the end of each day, data grids were compared for discrepancies; once any discrepancies, reference was made to the protocol in order to reach an agreement and eventually corrections were made. The aim of the review was to collect data on parameters that describe the type of sponsor, the origin (country) of the sponsor, the type of research, the type of investigators involved, the nationality and background of investigators, the site of research, the target population, the health domain and diseases under research. The schematic diagram below summarizes the data extraction process.
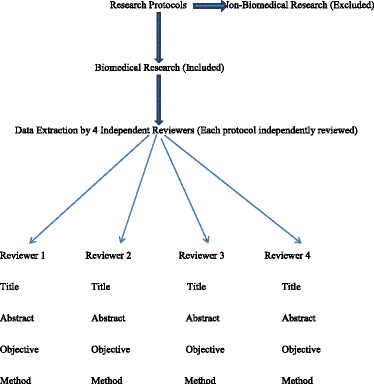


#### Data management

Data were entered into a Microsoft Access database and exported to EPIINFO and Microsoft Excel for analysis. Chi-square tests (chi2) were used to compare proportions; *p*-values determined the evidence of a significant difference between proportions and/or suggesting an association between them. Parameters were calculated at 95% confidence intervals. A p-value less than 5% (<0.05) suggested enough evidence to conclude that there was an association or a significant difference between proportions being compared.

Local sponsors were considered to be individuals, institutions, and organizations of Cameroonian origin, while international sponsors were considered individuals, institutions and organizations from abroad. Variables that were compared included; type of investigator, institution of investigators, study design, study site, Region of the study, health domain, target population and availability of a result dissemination plan.

## Results

**Fig. 1:**
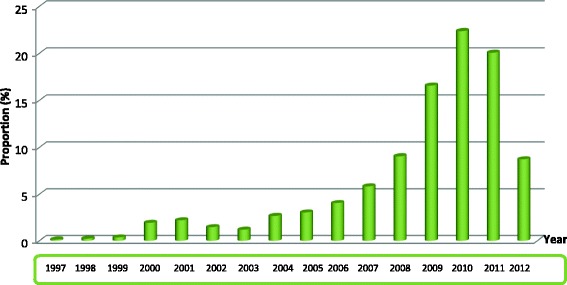
Overall distribution of protocols by year

**Fig. 2:**
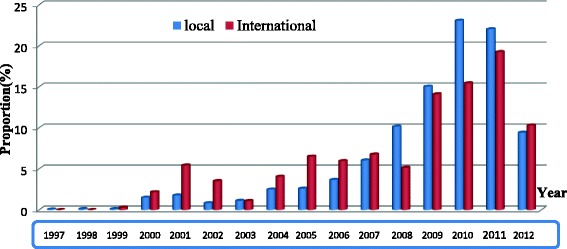
Distribution of protocols by year stratified by origin of sponsor (local versus international)

A total of 2172 protocols were reviewed. Figure 1 shows the rate of biomedical research in Cameroon over the years. One thousand eight hundred three (83.05%) were locally sponsored, and 369 (17.0%) were internationally sponsored. Most were hospital-based studies 1528 (72.4%). Based on the four domains of health interventions that have been adopted in the Cameroon national health strategy, 1323 (61.3%) were on “fight against diseases”; most of which were HIV 342 (25.8%) and Malaria 136 (10.3%). Most studies were concentrated in the Centre region 1242 (57.2%), while the least accessible and most vulnerable regions such as the Northern region hosted the least 15 (0.7%). There was enough evidence to suggest that the origin of sponsors (international or local) have an influence the choice of the study site (*p*-value = 0.01) and target population of research (*p*-value = 0.00) as seen on Fig. 2.

### Types of investigators

Student research projects were most common 1405 (64.7%), 1842 (87.8%) were of Cameroonian nationality, and 1223 (56.6) were males. From Table 1, 360 (52.3%) were medical doctors who have specialized, and most investigators1664 (77.1%) were affiliated with a university.

**Table 1:** Characteristics of investigators and their hosting institutions

Parameters	Tally	Frequency (%)
Sex		
Male	1223	56.6
Female	860	39.8
Unknown	89	4.10
Country of origin		
Cameroon	1842	87.8
Other	330	15.2
Professional background		
Specialist in Medicine	360	52.3
Pharmacist	13	1.9
Biologist	118	17.2
Public health	90	13.1
Others	107	15.6
Institution of investigator		
University	1664	77.1
Hospital	106	4.9
Pharmaceutical company	13	0.6
NGO	255	11.9
Others	96	4.5
Professional grade		
PhD	644	31.6
Masters	232	11.4
Undergraduate	108	5.3
Physician	1056	51.8

### Type and setting of research

Based on types of study designs that were used, 1890 (88.9%) were quantitative studies, 123 (5.8%) were qualitative studies and 113 (5.3%) were mixed (qualitative and quantitative). Observational studies accounted for 1550 (86.1%) while Interventional studies represented 250 (13.9%). From Table 2, hospital settings were the most preferred site to conduct research 1528 (72.4%).

**Table 2:** Types of study designs and study settings, stratified by student versus professional and by local versus international sponsor

Study Design stratified by student versus professional investigators			
	Students (%)	Professional (%)	*P*-value
Cross sectional	820 (56.4)	328 (45.7)	<0.001
Longitudinal	256 (17.6)	272 (37.9)	<0.001
Case control	71 (4.9)	29 (4.0)	0.36
Cohort	298 (20.5)	77 (10.7)	<0.001
Case series	4 (0.3)	4 (0.6)	0.31
Ecological studies	2 (0.2)	8 (1.2)	<0.001
Others	3 (0.2)	0	0.24
Total	1454	718	
Study Design stratified by local sponsors versus international sponsors			
	Local sponsor (%)	International (%)	
Cross sectional	844 (55.1)	109 (37.3)	<0.001
Longitudinal	320 (20.9)	135 (46.2)	<0.001
Case control	68 (4.4)	14 (4.8)	0.76
Cohort	285 (18.6)	31 (10.6)	<0.001
Case series	8 (0.5)	0	0.23
Ecological studies	5 (0.3)	3 (1.0)	0.09
Others	1 (0.1)	0	0.59
Total	1531	292	
Study Settings stratified by students versus professional			
	Students (%)	Professional (%)	
Laboratory	84 (6.1)	99 (13.5)	<0.001
Hospital	1104 (80.2)	424 (57.8)	<0.001
Community	126 (9.2)	176 (24.0)	<0.001
Other	63 (4.6)	35 (4.8)	0.84
Total	1377	734	
Study Settings stratified by local sponsor versus international sponsor			
	Local Sponsor (%)	International (%)	
Laboratory	128 (8.0)	43 (13.7)	<0.001
Hospital	1196 (75.0)	178 (56.5)	<0.001
Community	198 (12.4)	77 (24.4)	<0.001
Other	72 (4.5)	17 (5.4)	0.49
Total	1594	315	
Result Dissemination Plan			
Have a result dissemination plan	Local Sponsors	International Sponsors	*p*-value
Yes	137(7.6)	107 (29.0)	<0.001
No	1666 (92.4)	262 (71.0)	
Total	1803	369	

*P*-value <0.01: strong evidence; <0.05: good evidence

### Geographical distribution of research projects

There was a disproportionate distribution of biomedical research over the country. Most were concentrated in the Centre 1242 (57.2%), littoral 195 (9.0%) and the West regions 115 (5.3%) as presented in Table 3. Regions with the most vulnerable populations (those with greatest diseases related morbidity and mortality rates) were the least covered by biomedical research; these include the Adamawa 12(0.5%), East 14 (0.6%), and the North regions 15 (0.7%).

**Table 3:** Geographical distribution

Health Region	Number of Research projects (%)	Percentage of general population represented in 2012 (%)
Adamawa	12 (0.5)	5.3
Centre	1242 (57.2)	18.3
East	14 (0.6)	4.0
Far North	28 (1.3)	18.0
Littoral	195 (9.0)	14.9
North	15 (0.7)	10.9
North West	55 (2.5)	9.1
South	32 (1.5)	3.5
South West	44 (2.0)	7
West	115 (5.3)	9.0
Multiple regions	419 (19.3)	
Total	2172	20,386,800

Ref; INS online; https://www.citypopulation.de/Cameroon-Cities.html

### Research domains and target populations

A total of 1323 (61.3%) research projects targeted diseases of public health importance such as Malaria and HIV, 416 (19.3%) were focused on the health of mothers, children or adolescents. Men were the least target group 21 (1.0%), and 651 (30.8%) included participants from all age groups as seen in Table 4.

**Table 4:** Health domain and Target populations stratified by origin of sponsor

Health Domains stratified by origin of sponsor				
Health Domain	Total (%)	Local (%)	International (%)	*p*-value
Mother/child/Adolescent	416 (19.3)	377 (21.0)	23 (6.3)	<0.001
Fight against Diseases	1323 (61.3)	1084 (60.4)	249 (68.0)	0.01
Health promotion	170(7.9)	128 (7.1)	39 (10.6)	0.02
District Viability	60 (2.8)	45 (2.5)	14 (3.9)	0.13
Health determinants	191 (8.8)	160 (8.9)	41(11.2)	0.17
Total	2160 (100)	1794	366	
Target Populations stratified by origin of sponsor				
Target Population	Total	Local (%)	International (%)	*p*-value
Child	342 (16.2)	306 (17.3)	36 (10.1)	<0.001
Adolescent	74 (3.5)	60 (3.4)	10 (2.8)	0.57
Women	346 (16.3)	307 (17.4)	39 (10.4)	<0.001
Men	21 (1.0)	19 (1.1)	3 (0.9)	0.74
Adults	683 (32.3)	538 (30.5)	152 (43.2)	<0.001
All Ages	651 (30.8)	537 (30.4)	114 (32.5)	0.44
Total	2117 (100)	1766	351	

*P*-value <0.001: strong evidence; <0.05: good evidence

### Target diseases of the research

**Fig. 3:**
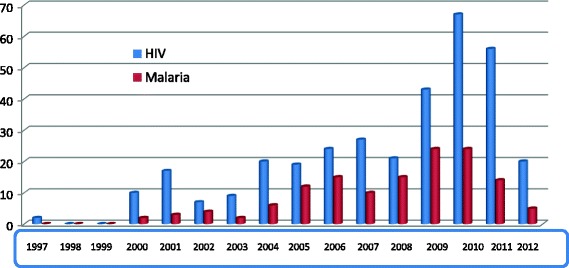
Trends of biomedical research on malaria and HIV by year

Table 5 summarized the main diseases that attracted research. They included HIV 342 (25.8%), Malaria 136 (10.3%) and neglected tropical diseases 126 (9.5%). Fig. 3 shows the trends of biomedical research on HIV/AIDS and Malaria. The figure indicates an increasing trend of research on HIV and Malaria over the years with peaks between 2009 and 2011.

**Table 5:** The most targeted diseases stratified by origin of sponsor

Diseases	Total (%)	Local sponsors (%)	International Sponsor (%)	*p*-value
Diabetes	101 (7.6)	90 (8.1)	11 (5.1)	0.13
HIV/AIDS	342 (25.8)	306 (27.6)	36 (16.7)	<0.001
Malaria	136 (10.3)	99 (8.9)	37 (17.2)	<0.001
Tuberculosis	44 (3.3)	39 (3.5)	5 (2.3)	0.37
Reproductive Health	33 (2.5)	29 (2.6)	4 (1.9)	0.55
Cancers	77 (5.8)	53 (4.8)	24 (11.2)	<0.001
Neurological/Mental Health	61 (4.6)	49 (4.4)	12 (5.6)	0.44
Cardiovascular Diseases	64 (4.8)	57 (5.1)	7(3.3)	0.26
Metabolic Diseases	37 (2.8)	33 (3.0)	4 (1.9)	0.37
Digestive System	54 (4.1)	51 (4.6)	3 (1.4)	0.03
Tropical/Infectious Diseases	126 (9.5)	107 (9.7)	19 (8.8)	0.68
Eye Diseases	19 (1.4)	15 (1.3)	4 (1.9)	0.49
Respiratory system diseases	38 (2.9)	29 (2.6)	9 (4.2)	0.20
STI/STD	17(1.3)	14(1.3)	3 (1.4)	0.91
Hepatitis	44 3.3)	35(3.20)	9 (4.2)	0.46
Others	130 (9.8)	102 (9.2)	28 (13.0)	0.09
Total	1323	1108	215	

*P*-value <0.01: strong evidence; <0.05: good evidence

### The influence of sponsors of biomedical research in Cameroon

The origin of sponsors (local or international) has an influence on the choice of the study site, population target, and type of disease. From Table 2, there was enough evidence to suggest that international sponsors promote mainly Laboratory and community-based studies (*p*-value <0.001) while local sponsors promote mainly hospital-based research (*p*-value <0.001). From Table 4, there was enough evidence to also suggest that international sponsors were more interested in disease-oriented research (*p*-value = 0.01) while local sponsors promoted research on mother, child and adolescents health (*p*-value <0.001). There were more children and women participants (*p*-value <0.001) in locally sponsored research as opposed to internationally sponsor research (*p*-value <0.001). Regarding types of diseases, Table 5 shows that international sponsors promoted research on malaria and cancers much more than local sponsors (*p*-value <0.001). Local sponsors promoted research on HIV (*p*-value <0.001) and digestive system diseases (*p*-value = 0.03) much more than international sponsors. There was enough evidence that international sponsors provide plans for result dissemination compared to local sponsors (*p*-value <0.001).

## Discussion

Biomedical research plays a primordial role to support and guide evidence-based health care policies that are necessary to enhance equity in the accessibility to health care and foster economic development [5, 2]. It is imperative that research findings reflect the health needs and health priorities of Cameroonians to ensure that appropriate health policies are elaborated inclusive of the most vulnerable and poorest populations, for an effective response to health challenges.

Following the adoption of the 2001–2015 [6] health sectors strategy plan, and the 2035 Development Vision [7], Cameroon has experienced an exponential increase in biomedical research. However, this study has demonstrated that biomedical research is not proportionately distributed around the country. Most research was concentrated in large, urban and easily accessible cities such as Yaoundé, while very few studies were conducted in less developed, hard-to-reach, and poorest areas, most of which are vulnerable with record numbers of disease burden and mortality. People in such locations have the least accessibility to primary health care and are poverty stricken. Examples include; the Far North, East, North, Adamawa, and the South. Our results were similar to that of a study conducted in 2010, at the Cameroon National Ethics Committee [8].

Most biomedical research protocols were student research projects. This was mainly thesis (dissertation) for obtaining graduate degrees from Universities. It is worth noting that findings from these students research projects are usually not considered by health authorities and stakeholders during decision making or policy development. The non-use of student research results by the health system clearly indicates a gap in the health information system. Also, there are no policies that promote collaboration between the health system, academia, and research institutions that can facilitate the flow of health information, knowledge, and promote the use of research findings by health authorities and policymakers.

Based on the 2010–2015 health sector strategy plans [6], the health system has been stratified into four main health domains (Table 4). In order to maintain an effective health system, there is a need for a balance to all components of the healthcare system. This study has shown that biomedical research does not adequately address all components of the health system. Over 80 % were focused on fighting against diseases (mainly HIV and Malaria) whereas only a few studies attempted to addressed components that would strengthen the system, such as health promotion and health service or health system development.

It is evident that Malaria and HIV are the leading causes of morbidity and mortality in Cameroon and in Sub-Saharan Africa [9–11]. Studies have shown that the Plasmodium parasite and the HIV virus are constantly undergoing genetic variation over the years [12–14], resulting in drug resistance and failures of some public health intervention programs. These justify why they have attracted funding from agencies, governments, research institutions, NGO’s and Academia. Unfortunately, only a little attention seems to have been given to other important components of the health system. While it is important for research to address eminent diseases, it is equally important for research to address the health service related components, to ensure that research-based policies are effectively implemented at the lowest level of the health system. We therefore believe that there is an absolute need to address every component of the health system adequately since all the components of a health system are related to one another for the health system to succeed.

Sponsors have a significant influence on the choice of the type of research, the disease to be studied, and the study setting or location. Most internationally sponsored studies were laboratories and community-based. Also, international sponsors were more interested in disease-oriented and health promotion research. On the other hand, local sponsors promoted hospital-based projects. One reason for this is that most of the locally sponsored studies were student research projects, which are most often self-funded. This explains the reasons for their choice in most cases as community-based studies are much more expensive to implement.

Women and children are considered to be the most vulnerable groups. Most health policies and health interventions target these two groups. However, this study has shown that children and women are less likely to be included as participants in research. International sponsors are less likely to promote the participation of women and children in research compared to local sponsors. The government needs to encourage the participation of women and children in research, to ensure that research guided policies are implemented in ways that may reflect their health needs.

## Conclusion

Biomedical research is on a rise in Cameroon. While most research is focused on diseases such as malaria and HIV, little attention is given to other components of the health system and to emerging diseases of the 21 century such as cardiovascular diseases, cancers, and neurological/mental health. There is a disproportionate distribution of biomedical research over the country. Most research projects are concentrated in urban cities (Centre, Littoral, and West), while the most vulnerable and less accessible populations are under-represented. There was enough evidence to suggest that the origin of sponsors have a significant influence on the type, site, target population, and target disease of biomedical research projects conducted in Cameroon.

To address these issues, we recommend that biomedical research must address all the components of the health system. The need for a strong collaboration between government, Ministry of Health and the National Ethics Committee is paramount to ensure that biomedical research is inclusive and responsive to health needs and to support appropriate health system reforms. We believe that these recommendations will go a long way to strengthen the health system, promote equity in health care access and promote economic development.

## Additional file

Additional file 1: Dataset. (XLSX 312 kb)

## Abbreviations

AIDS: Acquired immune deficiency syndrome
CI: Confidence interval
CNEC: Cameroon national ethics committee
HIV: Human immune deficiency virus
IRB: Institutional review board
NGO: Non-governmental organization
*P*-value: Degree of evidence of influence
STD: Sexually transmissible disease
STI: Sexually transmissible infection
